# Brimonidine Blocks Glutamate Excitotoxicity-Induced Oxidative Stress and Preserves Mitochondrial Transcription Factor A in Ischemic Retinal Injury

**DOI:** 10.1371/journal.pone.0047098

**Published:** 2012-10-09

**Authors:** Dongwook Lee, Keun-Young Kim, You Hyun Noh, Stephen Chai, James D. Lindsey, Mark H. Ellisman, Robert N. Weinreb, Won-Kyu Ju

**Affiliations:** 1 The Sophie and Arthur Laboratory for Optic Nerve Biology, Hamilton Glaucoma Center and Department of Ophthalmology, University of California San Diego, La Jolla, California, United States of America; 2 Research Institute of Clinical Medicine of Chonbuk National University-Biomedical Research Institute, Chonbuk National University Hospital, Jeonju, Jeonbuk, Republic of Korea; 3 Center for Research on Biological Systems, National Center for Microscopy and Imaging Research, and Department of Neuroscience, University of California San Diego, La Jolla, California, United States of America; Dalhousie University, Canada

## Abstract

Glutamate excitotoxicity-induced oxidative stress have been linked to mitochondrial dysfunction in retinal ischemia and optic neuropathies including glaucoma. Brimonindine (BMD), an alpha 2-adrenergic receptor agonist, contributes to the neuroprotection of retinal ganglion cells (RGCs) against glutamate excitotoxicity or oxidative stress. However, the molecular mechanisms of BMD-associated mitochondrial preservation in RGC protection against glutamate excitotoxicity-induced oxidative stress following retinal ischemic injury remain largely unknown. Here, we tested whether activation of alpha 2 adrenergic receptor by systemic BMD treatment blocks glutamate excitotoxicity-induced oxidative stress, and preserves the expression of mitochondrial transcription factor A (Tfam) and oxidative phosphorylation (OXPHOS) complex in ischemic retina. Sprague-Dawley rats received BMD (1 mg/kg/day) or vehicle (0.9% saline) systemically and then transient ischemia was induced by acute intraocular pressure elevation. Systemic BMD treatment significantly increased RGC survival at 4 weeks after ischemia. At 24 hours, BMD significantly decreased Bax expression but increased Bcl-xL and phosphorylated Bad protein expression in ischemic retina. Importantly. BMD significantly blocked the upregulations of *N*-methyl-D-aspartate receptors 1 and 2A protein expression, as well as of SOD2 protein expression in ischemic retina at 24 hours. During the early neurodegeneration following ischemic injury (12–72 hours), Tfam and OXPHOS complex protein expression were significantly increased in vehicle-treated retina. At 24 hours after ischemia, Tfam immunoreactivity was increased in the outer plexiform layer, inner nuclear layer, inner plexiform layer and ganglion cell layer. Further, Tfam protein was expressed predominantly in RGCs. Finally, BMD preserved Tfam immunoreactivity in RGCs as well as Tfam/OXPHOS complex protein expression in the retinal extracts against ischemic injury. Our findings suggest that systemic BMD treatment protects RGCs by blockade of glutamate excitotoxicity-induced oxidative stress and subsequent preservation of Tfam/OXPHOS complex expression in ischemic retina.

## Introduction

Glutamate excitotoxicity-induced oxidative stress has been linked to mitochondrial dysfunction in retinal ischemia and optic neuropathies including glaucoma [1]–[6], suggesting a distinct mitochondrial dysfunction-mediated cell death pathway is activated in retinal injury. Growing evidence indicates that glutamate excitotoxicity and/or oxidative stress is associated with mitochondrial DNA (mtDNA) damage-related mitochondrial dysfunction in retinal neurodegeneration [1], [5], [7], [8]. However, the molecular mechanisms underlying these effects are poorly understood.

Brimonidine (BMD), a selective alpha 2-adrenergic agonist that lowers intraocular pressure (IOP), protects retinal ganglion cells (RGCs) against glutamate excitotoxicity in culture system *in vitro* [9], [10] as well as in rodent models of experimental ischemia and glaucoma [11]–[15]. Its potential mechanisms include inhibition of glutamate release, upregulation of brain-derived neurotrophic factor expression, regulation of cytosolic Ca^2+^ signaling, and modulation of *N*-methyl-D-aspartate receptor (NMDAR, also as known as NR) function [12], [16], [17]. In addition, BMD is neuroprotective against oxidative stress that induces reactive oxygen species (ROS) formation including superoxide radicals (O_2_−) in culture system *in vitro* [14] as well as in rodent models of ocular hypertension [13], [18], [19]. Superoxide dismutases (SODs), cytosolic SOD1 and mitochondrial SOD2, are expressed in the ganglion cell layer (GCL) and inner plexiform layer (IPL) in rodent retina [20]. Emerging evidence suggests that SOD2 play a protective role against neuronal cell death that induced by glutamate excitotoxicity and oxidative stress [21].

**Figure 1:**
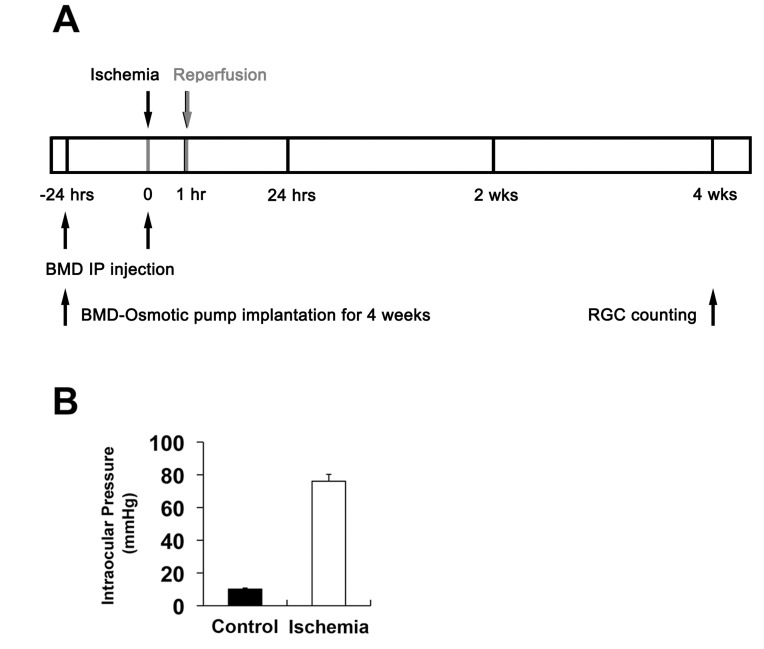
BMD treatment and induction of transient retinal ischemia. (A) Diagram for BMD administration before and after ischemic injury. BMD or vehicle were injected by IP for short terms or administrated systemically for 4 weeks using osmotic pumps. (B) IOP elevation in the rat eyes following transient ischemic injury. (B) Mean IOP was 76.1±4.2 mmHg during anterior chamber perfusion with saline. In contrast, mean IOP of contralateral control eyes was 11.2±1.7 mmHg (n = 10). BMD, brimonidine.

**Figure 2:**
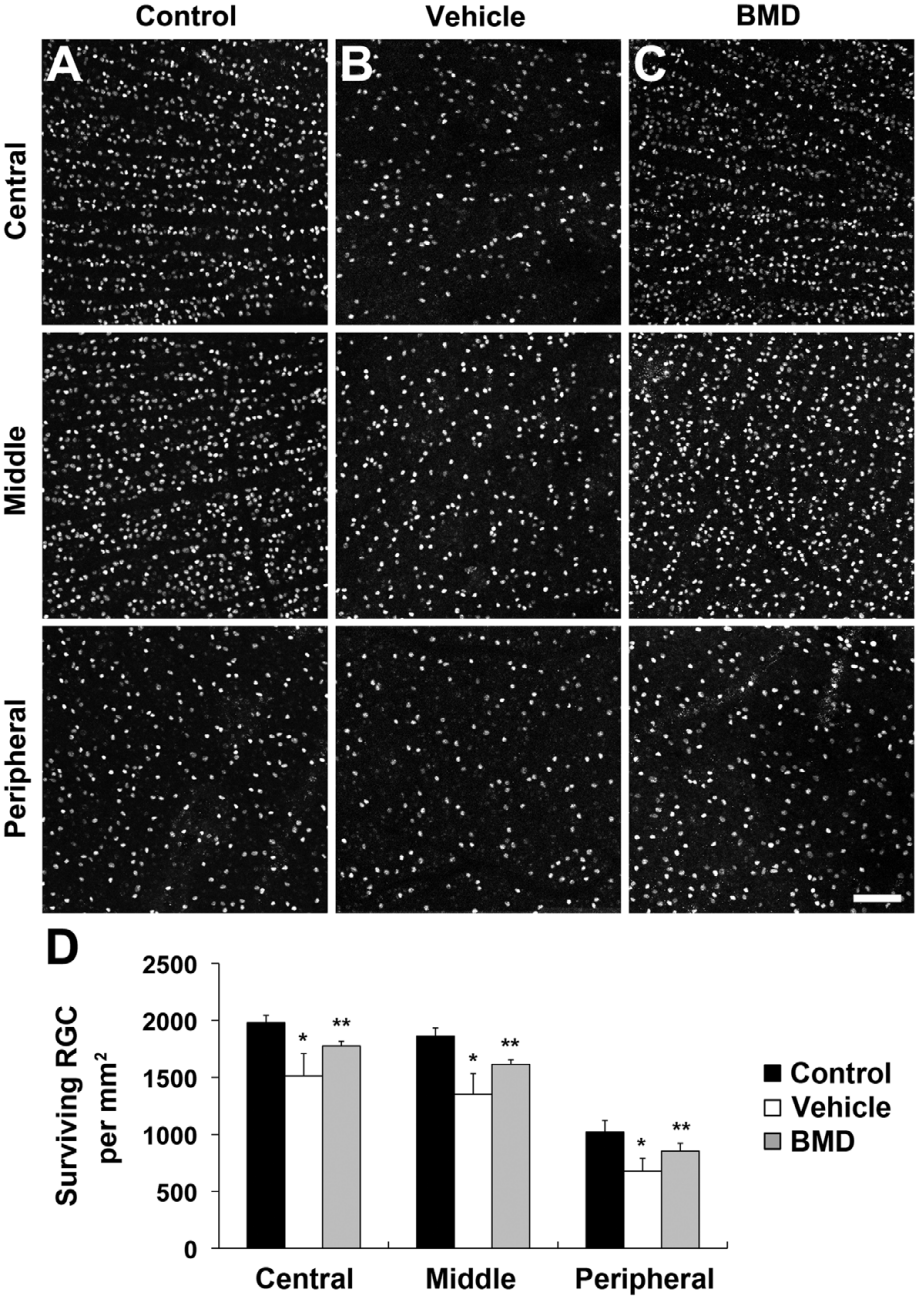
BMD-mediated protection of RGC survival in ischemic injury. BMD or vehicle were administrated systemically for 4 weeks using osmotic pumps. (A–C) Brn3a whole-mount immunohistochemistry. High magnification showed representative images from the middle area of retinas. In comparison with normal control retina (A), vehicle-treated ischemic retina showed greater RGC loss (B). In contrast, BMD significantly increased RGC survival in ischemic retina (C). (D) The quantitative analysis of RGC loss. Values are mean ± SD (n = 6 retinas/group). *Significant at p<0.05 compared with non-ischemic contralateral control retina or vehicle-treated ischemic retina. BMD, brimonidine. Scale bar = 100 µm.

Mitochondrial transcription factor A (Tfam, also as known as mtTFA), a nucleus encoded DNA-binding protein in mitochondria, has an important role in mitochondrial gene expression and mtDNA maintenance, and therefore is essential for oxidative phosphorylation (OXPHOS)-mediated ATP synthesis [22]–[25]. Mice lacking *Tfam* have impaired mtDNA transcription and loss of mtDNA that leads to bioenergetics dysfunction and embryonic lethality [22]. In contrast, overexpression of Tfam mediates delayed neuronal death following transient forebrain ischemia in mice [26]–[28] as well as neonatal hypoxic-ischemic brain injury rapidly increased Tfam and OXPHOS complex IV protein expression in a rat model, suggesting these responses may support endogenous repair mechanisms for mtDNA damage following hypoxic-ischemic brain injury [29].

Here, we tested whether activation of alpha 2 adrenergic receptor by systemic BMD treatment blocks glutamate excitotoxicity–induced oxidative stress and preserves the expression of Tfam and OXPHOS complex in ischemic retina.

**Figure 3:**
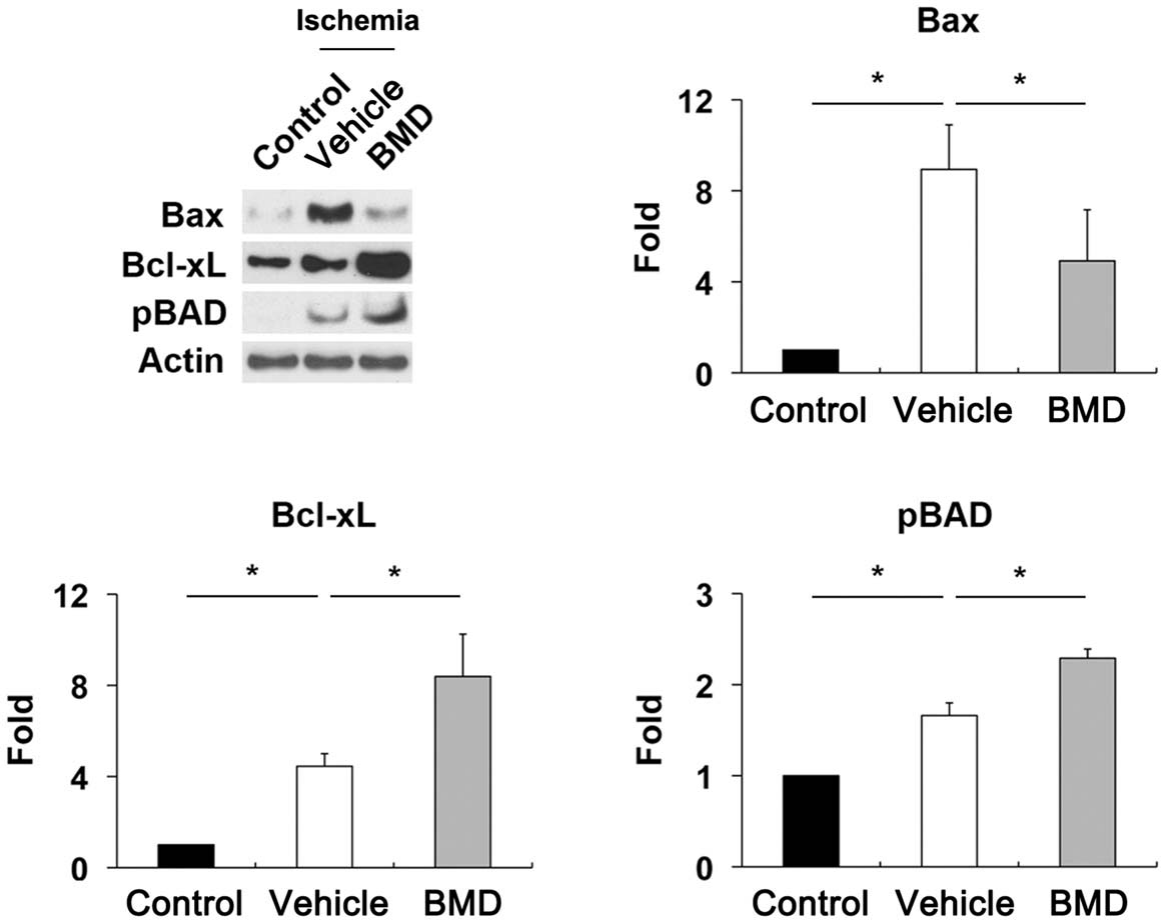
BMD-mediated blockade of apoptotic pathway. BMD or vehicle were injected once by IP at 24 hours before and at the time of initial IOP elevation for 24 hours after ischemia. Vehicle-treated ischemic retina significantly increased Bax, Bcl-xL, and pBad protein expression. In contrast, BMD significantly decreased Bax protein expression, but increased Bcl-xL and pBad protein expression compared with vehicle-treated ischemic retina. Values are mean ± SD (n = 4 retinas/group). *Significant at p<0.05 compared with non-ischemic contralateral control retina or vehicle-treated ischemic retina.

## Results

### Brimonidine Decreases Bax but Increases Bcl-xL and pBad Expression

The systemic treatment with BMD or vehicle began 24 hours before the induction of transient retinal ischemia and continued for 4 weeks post-ischemia (Figure 1 A). In addition, the rats received an IP injection of BMD or vehicle 24 hours prior to ischemia induction and another injection during the procedure. Transient retinal ischemia was induced by acute IOP elevation to 76.1±4.2 mmHg for 50 min during anterior chamber perfusion with saline (n = 10; Figure 1 B). The pressure was enough to induce retinal ischemia and the phenotype was similar to pathologic acute angle closure glaucoma because when IOP reached 60 mmHg, the retina flow rate decreased by 68% for retinal artery in rat [30]. The mean IOP of contralateral control eyes was 11.2±1.7 mmHg (n = 9; Figure 1 B). The alpha 2-adrenergic receptors are expressed in the somas of the cells in the GCL and inner nuclear layer (INL) [11]. To quantify RGC survival following BMD treatment in ischemic retina, we performed whole-mount immunohistochemistry for Brn3a antibody. In comparison with control retina (Figure 2 A and D), vehicle-treated ischemic retinas showed about 27% of RGC loss at 4 weeks after transient ischemia, (p<0.05; Figure 2 B and D). In contrast, BMD treatment significantly increased RGC survival by an approximate 20% compared to vehicle-treated ischemic retina (p<0.05; Figure 2 C and D).

**Figure 4:**
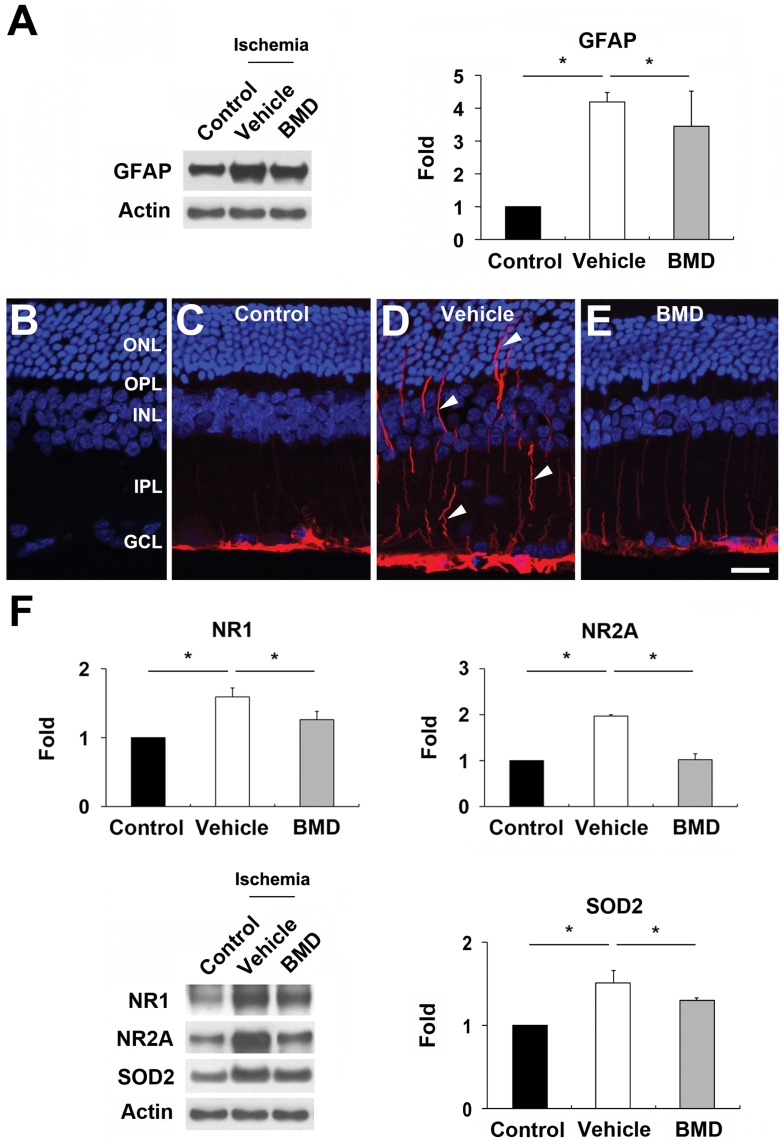
BMD-mediated blockade of the upregulations of GFAP, NMDA receptors and SOD2 expression in ischemic retina. BMD or vehicle were injected once by IP at 24 hours before and at the time of initial IOP elevation for 24 hours after ischemia. (A) Vehicle-treated ischemic retina significantly increased GFAP protein expression compared with control retina. In contrast, BMD significantly decreased GFAP protein expression in ischemic retina. (B–E) GFAP immunohistochemisty. When the primary antibody for GFAP was omitted, there was no labeling of the secondary antibody (B). In comparison with control retina (C), vehicle-treated ischemic retina showed activation of müller cells (arrowheads) and astrocytes (D). In contrast, BMD significantly decreased activation of müller cells and astrocytes (E). ONL, outer nuclear layer; OPL, outer plexiform layer; INL, inner nuclear layer; IPL, inner plexiform layer; GCL, ganglion cell layer. Scale = 20 µm. (F) Vehicle-treated ischemic retina significantly increased NR1, NR2A and SOD2 protein expression compared with control retina. In contrast, BMD significantly decreased NR1, NR2A and SOD2 protein expression in ischemic retina. Values are mean ± SD (n = 4 retinas/group). *Significant at p<0.05 compared with non-ischemic contralateral control retina or vehicle-treated ischemic retina.

To determine whether BMD modulates apoptotic cell death pathway in ischemic retina, we performed Western blot analysis using antibodies for Bax, Bcl-xL and phosphorylated Bad (pBad). We found that Bax protein expression was significantly increased in vehicle-treated ischemic retina by 8.94±1.96-fold compared with control (p<0.05). In contrast, BMD treatment significantly decreased Bax expression by 4.92±2.24-fold in ischemic retina (p<0.05; Figure 3). In comparison with control, vehicle-treated ischemic retina significantly increased Bcl-xL and pBad protein expression by 1.66±0.14- and 4.46±0.55-fold, respectively (p<0.05; Figure 3). Intriguingly, BMD treatment showed greater increases of Bcl-xL and pBad protein expression by 2.29±0.1- and 8.4±1.86-fold in ischemic retina, respectively (p<0.05; Figure 3).

**Figure 5:**
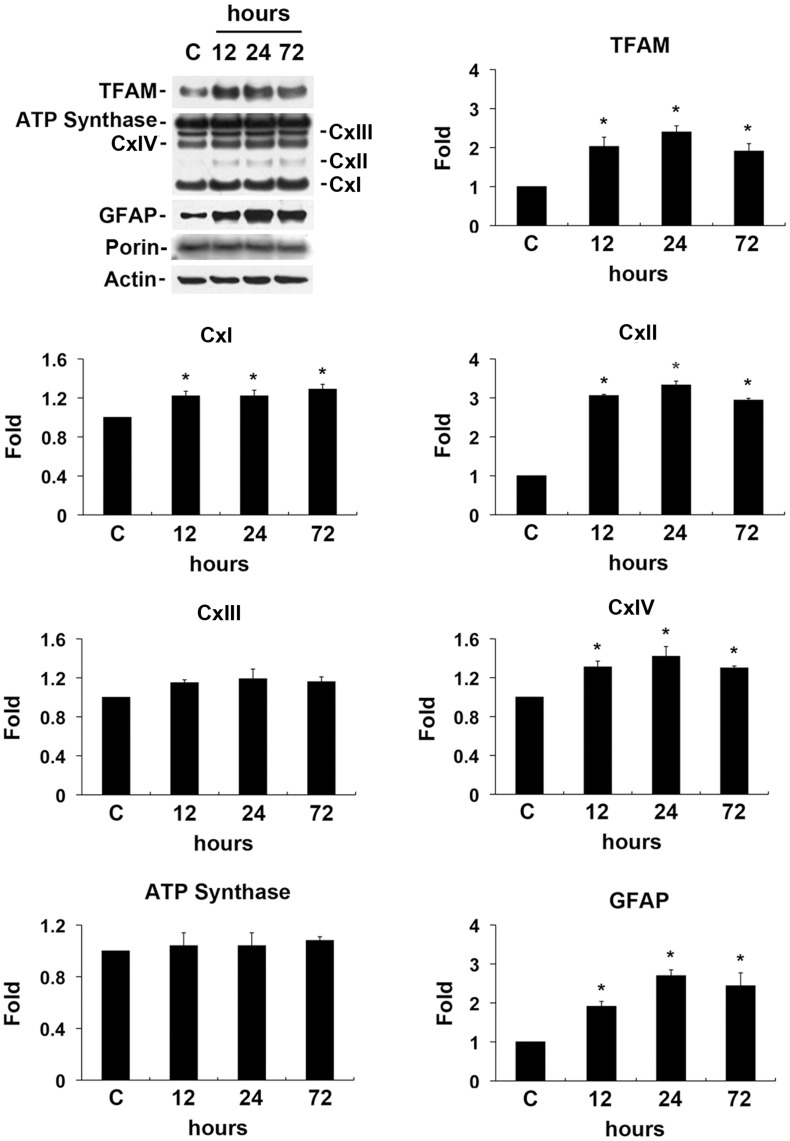
Alterations of Tfam and OXPHOS complex protein expression in ischemic retina. BMD or vehicle were injected once by IP at 24 hours before and at the time of initial IOP elevation and administrated intraperitoneally for 72 hours after ischemia. Acute IOP elevation significantly increased GFAP, Tfam and OXPHOS complex (I, II, and IV), but did not change OXPHOS complex III and ATP synthase protein expression in ischemic retina. Values are mean ± SD (n = 4 retinas/group). *Significant at p<0.05 compared with non-ischemic contralateral control retina. Cx, Complex.

### Brimonidine Blocks the Upregulation of GFAP, NMDA Receptors and SOD2 Expression

We investigated whether BMD blocks the upregulations of glial fibrillary acidic protein (GFAP), NR1 and NR2A, and SOD2 expression in ischemic retina. Astroglia and/or müller cells activation coincides with RGC degeneration in the hypertensive retina of the human, rat or mouse [3], [31]–[33]. As shown in Figure 4, vehicle-treated ischemic retina significantly increased GFAP protein expression by 4.20±0.30-fold compared with control. In contrast, BMD treatment significantly decreased GFAP protein expression in ischemic retina at 24 hours (p<0.05; Figure 4 A). When the primary antibody was omitted, as a control for GFAP immunohistochemistry, there was no labeling by the secondary antibody in control retina (Figure 4 B). Compared with control retina (Figure 4 C), GFAP immunoreactivity was increased in müller cells and astrocytes of the nerve fiber layer of vehicle-treated ischemic retina at 24 hours (Figure 4 D). In contrast, BMD treatment decreased GFAP immunoreactivity in ischemic retina (Figure 4 E). NR1, NR2A and SOD2 protein expression were significantly increased by 1.59±0.13-, 1.96±0.03-, and 1.51±0.15-fold, respectively, compared with control retina (p<0.05). In contrast, BMD treatment significantly decreased NR1, NR2A and SOD2 protein expression in ischemic retina (p<0.05; Figure 4 F).

**Figure 6:**
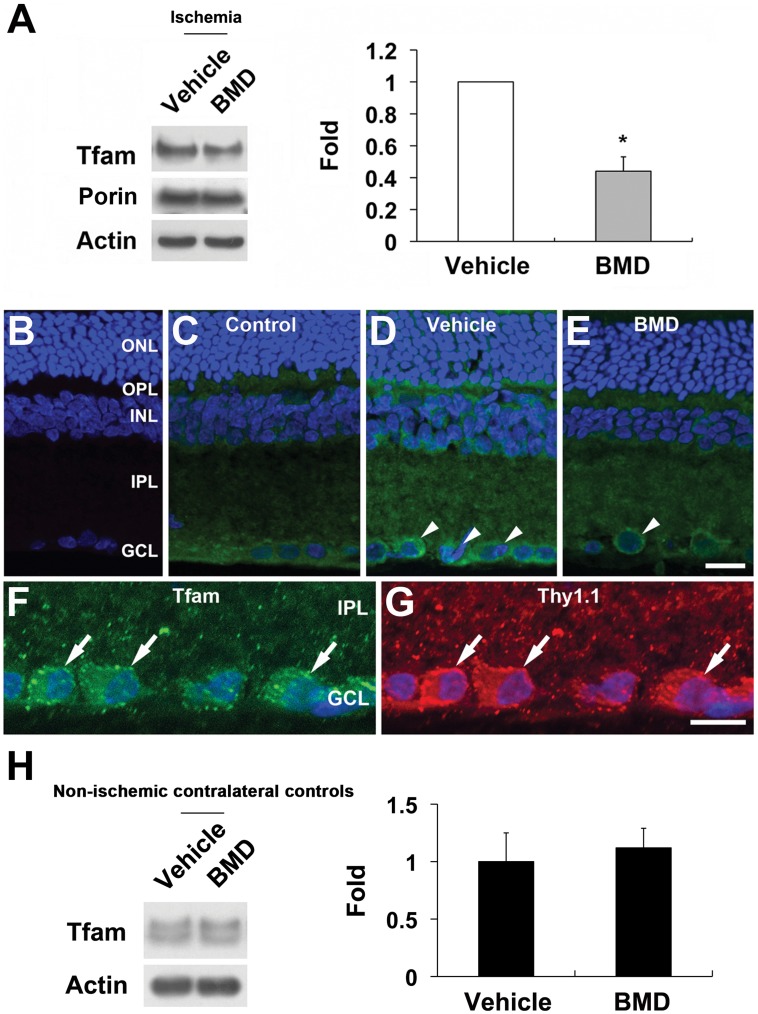
BMD-mediated restoration of Tfam protein expression in ischemic retina. BMD or vehicle were injected once by IP at 24 hours before and at the time of initial IOP elevation for 24 hours after ischemia. (A) BMD significantly decreased Tfam protein expression compared with vehicle-treated ischemic retina. Values are mean ± SD (n = 4 retinas/group). *Significant at p<0.05 compared with vehicle-treated ischemic retina. *Significant at p<0.05 compared with vehicle-treated ischemic retina. (B–E) Tfam immunohistochemisty. When the primary antibody for Tfam was omitted, there was no labeling of the secondary antibody (B). In comparison with control retina (C), vehicle-treated ischemic retina increased Tfam protein expression in the OPL, INL, IPL, and GCL. Note that Tfam immunoreactivity was increased in neurons in the GCL (arrowheads) (D). In contrast, BMD significantly decreased in neurons in the GCL (arrowhead) (E). (F and G) Tfam and Thy1.1 (red) double labeling. Neurons containing Tfam immunoreactivity were colabeled with Thy1.1, a marker for RGCs (arrows), indicating that RGCs contained Tfam protein. (H) There was no significant difference between vehicle- and BMD-treated non-ischemic contralateral control retinas (p = 0.5). ONL, outer nuclear layer; OPL, outer plexiform layer; INL, inner nuclear layer; IPL, inner plexiform layer; GCL, ganglion cell layer; BMD, brimonidine. Scale bar = 20 µm.

### Brimonidine Preserves Tfam and OXPHOS Complex Protein Expression

To determine whether ischemic injury alters protein expression for GFAP, Tfam and OXPHOS complex, we performed Western blot analysis. Increases in GFAP, Tfam, and OXPHOS complex (II and IV) protein expression were maximal 24 hours later by 2.7±0.15-, 2.4±0.16-, 3.33±0.3-, and 1.42±0.1-fold in ischemic retina, respectively (p<0.05; Figure 5). The relative concentration of each of these proteins expression was less at 72 hours than at 24 hours after ischemia-reperfusion. In addition, OXPHOS complex I protein was significantly increased up to 72 hours (p<0.05; Figure 5). However, there was no difference in ATP synthase protein expression. These results suggest that ischemic injury triggers the upregulation of Tfam and OXPHOS complex protein expression in the early neurodegenerative events in the retina.

**Figure 7:**
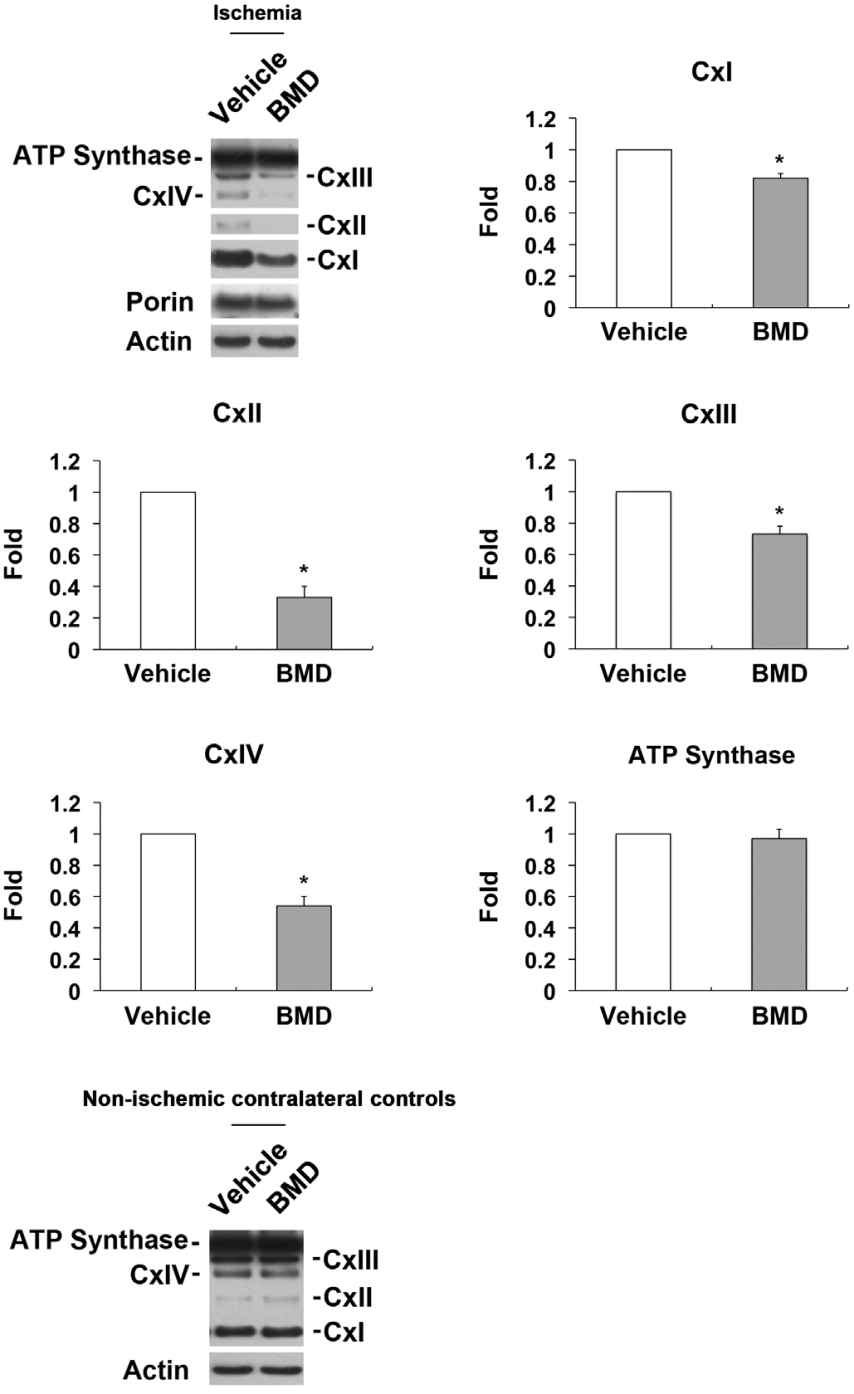
BMD-mediated restoration of OXPHOS complex protein expression in ischemic retina. BMD or vehicle were injected once by IP at 24 hours before and at the time of initial IOP elevation for 24 hours after ishcemia. Note that BMD significantly decreased OXPHOS complex (I, II, III and IV) but did not change ATP synthase protein expression in ischemic retina. Values are mean ± SD (n = 4 retinas/group). There was no significant difference between vehicle- and BMD-treated non-ischemic contralateral control retinas. *Significant at *P*<0.05 compared with vehicle-treated ischemic retina. Cx, Complex; BMD, brimonidine.

BMD treatment preserved Tfam protein expression at 24 hours (p<0.05; Figure 6 A) compared with vehicle-treated ischemic retina. When the primary antibody was omitted, as a control for Tfam immunohistochemistry, there was no labeling by the secondary antibody in control retina (Figure 6 B). Compared with control retina (Figure 6 C), Tfam immunoreactivity was increased in the outer plexiform layer (OPL) and IPL, as well as in neurons of the GCL in vehicle-treated ischemic retina at 24 hours (Figure 6 D). In contrast, Tfam expression in the OPL, IPL and GCL (Figure 7 E) of BMD-treated ischemic retina was similar to control retinas. To confirm whether RGCs express Tfam, RGCs were co-immunostained with Tfam and Thy1.1, a specific marker for RGCs. Tfam was expressed predominantly in GCL neurons that were positive for the RGC protein Thy1.1 (Figure 6 F and G). In addition, there was no significant difference of Tfam expression between vehicle- and BMD-treated non-ischemic control retinas (p = 0.5; Figure 6 H), suggesting that BMD did not directly affect expression of Tfam protein in control. Further, Western blot analyses showed that BMD treatment also preserved OXPHOS complex (I, II, III and IV) protein expression (p<0.05; Figure 7). However, there was no difference in ATP synthase protein expression at 24 hours compared with vehicle-treated ischemic retina (Figure 7).

## Discussion

Consistent with our results that BMD promoted RGC survival against ischemic injury, we found that BMD significantly decreased Bax but increased Bcl-xL and pBad protein expression in ischemic retina. Bax is a pro-apoptotic member of the Bcl-2 family that is essential in many pathways of apoptosis [34], [35] as well as directly interacts with the component forming the mitochondrial permeability transition pore that allows proteins to escape from the mitochondria into cytosol to initiate apoptosis [36], [37], [38]. Bax is counteracted by Bcl-xL that forms heterodimers with dephosphorylation of Bad, which inactivates Bcl-xL and pBad eliminates this dimerization, which activates Bcl-xL [39], [40]. BMD-mediated activation of alpha 2 receptors has been reported to activate the phosphatidyl inositol-3-kinase (PI3K) and protein kinase/Akt (Akt) pathways that increase survival factors including Bcl-2 and Bcl-xL [41]. Further, Bcl-xL promotes mitochondrial adenine-nucleotide exchange and prevents mitochondrial hyperpolarization by maintaining mitochondrial membrane permeability [42], [43]. Together with these findings, our results suggest that BMD promotes RGC survival against mitochondrial damage-mediated apoptotic pathway in ischemic injury by increasing Bcl-xL and pBad expression. These results reflect the possibility that increased Bcl-xL and pBad expression may represent endogenous repair mechanism against apoptotic pathway and BMD may contribute to the blockade of Bax-mediated increase of mitochondrial membrane permeability or the promotion of mitochondrial homeostasis in ischemic retinal injury.

Glutamate excitotoxicity and/or oxidative stress has been linked to mitochondrial dysfunction in many neurodegenerative disorders including retinal ischemia and glaucoma [2], [44]–[47]. Growing evidence indicates that BMD is neuroprotective against glutamate excitotoxicity or oxidative stress in retinal ischemia and experimental glaucoma [12]–[14], [19]. Importantly, it has been proposed that BMD may protect RGCs by inhibiting glutamate release, modulating NMDA receptor function or interfering free oxygen radicals [12], [14], . In the current study, our findings demonstrated the first evidence that BMD significantly blocks the upregulation of NR1 and NR2A protein expression in ischemic retina, suggesting the possibility that inhibition of glutamate release by BMD-mediated alpha 2-adrenergic receptor activation may block the upregulation of NMDA receptor expression and ameliorate glutamate excitotoxicity-mediated neurodegeneration in ischemic retina. Because MK801, a NMDA receptor antagonist, activates PI3K/Akt pathway, reduces Bad expression, and subsequently protects RGCs in ischemic retina [48], our findings suggest that BMD may contribute to RGC survival through activation of pro-survival PI2K/Akt pathway by not only activation of alpha 2-adrenergic receptor, but also blockade of glutamate excitotoxicity in ischemic retina. Thus, the potential mechanisms underlying these synergic effects of BMD on ischemic retina should be further explored.

We observed that BMD blocks the upregulation of SOD2 protein expression in ischemic retina. Because overexpression of SOD2 contributes to the reduction of mitochondrial superoxide level, protection of mitochondrial morphology and functions, and mitochondrial resistance against glutamate excitotoxicity-induced oxidative cytotoxicity [21], our result indicates that increasing SOD2 expression may play a critical role in the mitochondria as a compensatory mechanism for protecting RGCs against glutamate exicitotoxicity-induced oxidative stress in ischemic retina. Moreover, BMD-mediated preservation of mitochondrial SOD2 expression may provide an important neuroprotective implication for ameliorating oxidative stress in ischemic retina. In addition, we found that BMD blocks increased GFAP expression in müller cells in ischemic retina. Although further studies need to be investigated, it is possible that BMD-mediated protection may indirectly modulate glial reaction against pressure-induced ischemic insults. Thus, we believe that BMD has therapeutic potentials for treating glutamate excitotoxicity- and/or oxidative stress-mediated neurodegenerative disorders including retinal ischemia and glaucoma.

Ischemic injury triggers mitochondrial dysfunction including ROS, mtDNA damage, or OXPHOS impairment in the central nervous system [29], [49]. Although mtDNA is particularly susceptible to glutamate excitotoxicity-induced oxidative stress [44], it is unknown whether glutamate excitotoxicity-induced oxidative stress can directly trigger mtDNA dysfunction in ischemic retina. Tfam regulates mtDNA copy numbers in mammals and the levels of Tfam correlate with the levels of mtDNA [25], [50]. Emerging evidence suggests that the Tfam and OXPHOS complex IV are rapidly increased in the early neurodegenerative events of neonatal hypoxic-ischemic brain injury, suggesting that Tfam may contribute to endogenous repair mechanism of injured brain neurons [29]. Further, recent studies reported that overexpression of Tfam protect mitochondria against β–amyloid-induced oxidative damage in human SH-SY5Y neuroblastoma cells and ameliorates delayed neuronal cell death in the hippocampus following transient forebrain ischemia in mice [26], [27]. On the other hand, in mice lacking *Tfam* there is impaired mtDNA transcription and mtDNA loss is triggered, therefore leading to mitochondrial bioenergetic dysfunction-mediated embryonic lethality [22]. However, it is unknown that whether ischemic injury alters Tfam/OXPHOS complex protein expression in the retina and whether BMD blocks this alteration of Tfam/OXPHOS protein expression in ischemic retina.

In the current study, we observed that retinal ischemic injury triggers significant increase of Tfam/OXPHOS complex protein expression in the early neurodegenerative events (12–72 hours) and BMD preserves Tfam/OXPHOS complex (I-IV) protein expression in ischemic retina. Consistent with these results, we also found that Tfam immunoreactivity is preserved in RGCs to the control level following ischemic injury. These results suggest that increasing Tfam/OXPHOS complex expression may be an important mitochondria-related compensatory response against glutamate excitotoxicity-induced oxidative stress in ischemic retina. Further, BMD-mediated preservation of Tfam/OXPHOS complex may provide a potential mechanism for protecting RGCs against mitochondrial dysfunction-mediated ischemic retinal injury. Future studies will investigate the precise molecular mechanism underlying BMD-mediated mtDNA preservation in ischemic retina or other optic neuropathies including glaucoma.

These results provide the evidence that systemic BMD treatment may contribute to RGC survival by blockade of glutamate exicitotoxicity-induced oxidative stress in ischemic retina. Moreover, the preservation of Tfam/OXPHOS complex expression by BMD may provide an important therapeutic potential for protecting RGCs against mitochondrial dysfunction induced by glutamate excitotoxicity and/or oxidative stress in ischemic retina. Thus, our findings suggest that the potential mechanisms underlying these effects by BMD may provide new therapeutic strategies for ameliorating mitochondrial dysfunction-mediated neurodegeneration in ischemic injury.

## Materials and Methods

### Animals

Female, Sprague-Dawley rats (250–300 g in weight; Harlan Laboratories, Indianapolis, IN) were housed in covered cages, fed with a standard rodent diet ad libitum, and kept on a 12 hours light/12 hours dark cycle. All procedures concerning animals were performed in accordance with the ARVO statement for the Use of Animals in Ophthalmic and Vision Research and under protocols approved by institutional IACUC committees at the University of California San Diego.

### Induction of Transient Retinal Ischemia

The rats were anesthetized with a mixture of ketamine (50 mg/kg, Ketaset; Fort Dodge Animal Health, Fort Dodge, IA) and xylazine (5 mg/kg, TranquiVed; Vedeco, Inc., St. Joseph, MO) by intraperitoneal (IP) injection. Eyes were also treated with 1% proparacaine drops. A 30-gauge needle was inserted into the anterior chamber of right eye that was connected by flexible tubing to a saline reservoir. By raising the reservoir, IOP was elevated to 70–80 mmHg for 50 minutes. Shame treatment was performed in the contralateral eyes by the insertion of a needle in the anterior chamber without saline injection. Retinal ischemia was confirmed by observing whitening of the iris and loss of the retina red reflex. IOP was measured with a tonometer (TonoLab; Tiolatoy, Helsinki, Finland) during ischemia. Non-ischemic contralateral control retinas were used as a control.

### Pharmacologic Treatment

BMD was purchased from United States Biological (USBiological, Swampscott, MA). Two groups of rats, which were randomly divided, were studied after transient retinal ischemia: one group was treated with vehicle (0.9% saline, n = 13 rats), and the other group was treated with BMD (1 mg/kg/day, n = 13 rats). BMD or vehicle were injected once by IP at 24 hours before and at the time of initial IOP elevation for short term experiments, as well as administrated systemically for 4 weeks using osmotic pumps (Alzet; Durect, Cupertino, CA) that were implanted subcutaneously on the back at 24 hours before transient retinal ischemia.

### Tissue Preparation

Twelve to 72 hours after acute IOP elevation, light adapted rats were anesthetized with IP injection of mixture of ketamine/xylazine, as described, and then rats were killed by CO_2_ inhalation. The rats were then perfused transcardially with 0.9% saline followed by 4% paraformaldehyde in 1X phosphate buffer saline (PBS, pH 7.4). Both eyes enucleated and fixed in 4% paraformaldehyde in PBS for 4 hours at 4°C. After several washes in PBS, the retinas were dissected and then dehydrated through graded ethanols and embedded in polyester wax, as described previously [51]. For Western blot analysis, dissected retinas were used immediately.

### Whole-mount Immunohistochemical Analysis

Retinas from enucleated eyes were dissected as flattened whole-mounts at 4 weeks after transient retinal ischemia. Retinas were immersed in PBS containing 30% sucrose for 24 hours at 4°C. The retinas were blocked in PBS containing 3% donkey serum, 1% bovine serum albumin, 1% fish gel and 0.1% triton X-100, and incubated with polyclonal goat anti-Brn3a antibody (1∶500; Santa Cruz Biotechnology, Santa Cruz, CA) for 72 hours at 4°C. After several wash steps, the retinas were incubated with the secondary antibody, Alexa Fluor-568 donkey anti-goat IgG antibody (Invitrogen) for 24 hours, and subsequently washed with PBS. Images were captured with a spinning-disc confocal microscope (Olympus America Inc., Center Valley, PA) equipped with a high-precision closed loop XY stage and closed loop Z control with commercial mosaic acquisition software (MicroBrightField; MBF Bioscience Inc., Williston, VT). The microscope is equipped with high-resolution high-sensitive CCD camera for high-speed mosaic acquisition. To count RGCs, each retinal quadrant was divided into three zones by central, middle, and peripheral retina (one sixth, three sixths, and five sixths of the retinal radius). RGC densities were measured in 16 distinct areas of 0.344 mm^2^ (two areas at central, middle, and peripheral per retinal quadrant) per condition by two investigators in a masked fashion, and the scores were averaged.

### Immunohistochemical Analysis

Immunohistochemical staining of 7 µm wax sections of full thickness retina were performed as previously described [51]. Five sections per wax block from each group were used for immunohistochemical analysis. Primary antibodies were monoclonal mouse anti-GFAP antibody (1∶500; Sigma, St. Louis, MO), polyclonal goat anti-Tfam antibody (1∶100; Santa Cruz Biotechnology) and monoclonal mouse anti-Thy1.1 antibody (Clone OX-7, 1∶500; Millipore, Billerica, MA). To prevent non-specific background, tissues were incubated in 1% bovine serum albumin/PBS for 1 hour at room temperature before incubation with the primary antibodies for 16 hours at 4°C. After several wash steps, the tissues were incubated with the secondary antibodies, FITC-conjugated donkey anti-goat IgG antibody (Invitrogen, Carlsbad, CA), Alexa Fluor 568 dye-conjugated goat anti-mouse IgG antibody (Invitrogen) or Alexa Fluor 488 dye-conjugated anti-rabbit IgG antibody (1∶100; Invitrogen) for 4 hours at 4°C and subsequently washed with PBS. The sections were counterstained with the nucleic acid stain Hoechst 33342 (Invitrogen) in PBS. Images were acquired with confocal microscopy (Olympus FluoView1000; Olympus, Tokyo, Japan).

### Western Blot Analysis

The retinas were homogenized in a glass-Teflon Potter homogenizer in lysis buffer (20 mM HEPES [pH 7.5], 10 mM KCl, 1.5 mM MgCl_2_, 1 mM EDTA, 1 mM EGTA, 1 mM DTT, 0.5% CHAPS, and complete protease inhibitors; Roche Biochemicals, Indianapolis, IN). Each sample (10 µg; n = 3 retinas/groups) was separated by PAGE and electrotransferred to polyvinylidenedifluoride membrane. The membrane was blocked with 5% nonfat dry milk and 0.1% Tween-20 in PBS, incubated with monoclonal mouse anti-GFAP (1∶3000; Sigma), goat polyclonal anti-Tfam antibody (1∶1000; Santa Cruz Biotechnology), mouse monoclonal anti-total OXPHOS complex antibody (containing a mixture of antibodies to COXI-IV and ATP synthase, 1∶3000; Invitrogen), rabbit polyclonal anti-Bax antibody (1∶500; Santa Cruz Biotechnology), rabbit polyclonal anti-Bcl-xL antibody (1∶1000; Cell Signaling, Danvers, MA), mouse monoclonal anti-phosphorylated Bad (pBad, 1∶2000; Cell Signaling), mouse monoclonal anti-NR1 antibody (1∶1000; BD Pharmingen, San Diego, CA), rabbit polyclonal anti-NR2A antibody (1∶500; Millipore), rabbit polyclonal anti-voltage-dependent anion channel (VDAC) antibody (Porin, 1∶1000; Calbiochem, La Jolla, CA) and mouse monoclonal anti-actin antibody (1∶5000, Millipore, Billerics, MA). After several washes in Tween/PBS, the membranes were incubated with peroxidase-conjugated donkey anti-goat IgG (1∶5000: Bio-Rad, Hercules, CA, USA), goat anti-rabbit IgG (1∶5000; Bio-Rad) or goat anti-mouse IgG (1∶5000; Bio-Rad), and developed using chemiluminescence detection (ECL Plus; GE Healthcare Bio-Science, Piscataway, NJ). The scanned film Images were analysed by ImageJ (http://rsb.info.nih.gov/ij/) and band densities were normalized to the band densities for actin or porin.

### Statistical Analysis

Data were presented as the mean ± SD. Comparison of two or three experimental conditions was evaluated using the unpaired, two-tailed Student’s *t*-test or one-way analysis of variance and the Bonferroni *t*-test. p<0.05 was considered to be statistically significant.

## References

[1] Jarrett SG, Lin H, Godley BF, Boulton ME (2008) Mitochondrial DNA damage and its potential role in retinal degeneration. Prog Retin Eye Res 27: 596–607. 18848639 10.1016/j.preteyeres.2008.09.001

[2] Ju WK, Lindsey JD, Angert M, Patel A, Weinreb RN (2008) Glutamate receptor activation triggers OPA1 release and induces apoptotic cell death in ischemic rat retina. Mol Vis 14: 2629–2638. 19122832 PMC2613079

[3] Park SW, Kim KY, Lindsey JD, Dai Y, Heo H, et al (2011) A selective inhibitor of drp1, mdivi-1, increases retinal ganglion cell survival in acute ischemic mouse retina. Invest Ophthalmol Vis Sci 52: 2837–2843. 21372007 10.1167/iovs.09-5010 PMC3088566

[4] Nguyen D, Alavi MV, Kim KY, Kang T, Scott RT, et al (2011) A new vicious cycle involving glutamate excitotoxicity, oxidative stress and mitochondrial dynamics. Cell Death Dis 2: e240. 22158479 10.1038/cddis.2011.117 PMC3252734

[5] Chan AS, Saraswathy S, Rehak M, Ueki M, Rao NA (2012) Neuroglobin protection in retinal ischemia. Invest Ophthalmol Vis Sci 53: 704–711. 22167093 10.1167/iovs.11-7408 PMC3317415

[6] Osborne NN (2008) Pathogenesis of ganglion “cell death” in glaucoma and neuroprotection: focus on ganglion cell axonal mitochondria. Prog Brain Res 173: 339–352. 18929120 10.1016/S0079-6123(08)01124-2

[7] Lee S, Van Bergen NJ, Kong GY, Chrysostomou V, Waugh HS, et al (2011) Mitochondrial dysfunction in glaucoma and emerging bioenergetic therapies. Exp Eye Res 93: 204–212. 20691180 10.1016/j.exer.2010.07.015

[8] Kong YX, Van Bergen N, Trounce IA, Bui BV, Chrysostomou V, et al (2011) Increase in mitochondrial DNA mutations impairs retinal function and renders the retina vulnerable to injury. Aging Cell 10: 572–583. 21332926 10.1111/j.1474-9726.2011.00690.x

[9] Ogidigben M, Chu TC, Potter DE (1994) Alpha-2 adrenoceptor mediated changes in aqueous dynamics: effect of pertussis toxin. Exp Eye Res 58: 729–736. 7925712 10.1006/exer.1994.1070

[10] Toris CB, Camras CB, Yablonski ME (1999) Acute versus chronic effects of brimonidine on aqueous humor dynamics in ocular hypertensive patients. Am J Ophthalmol 128: 8–14. 10482088 10.1016/s0002-9394(99)00076-8

[11] Wheeler LA, Gil DW, WoldeMussie E (2001) Role of alpha-2 adrenergic receptors in neuroprotection and glaucoma. Surv Ophthalmol 45 Suppl 3: S290–294; discussion S295–296. 10.1016/s0039-6257(01)00206-5 11377451

[12] Dong CJ, Guo Y, Agey P, Wheeler L, Hare WA (2008) Alpha2 adrenergic modulation of NMDA receptor function as a major mechanism of RGC protection in experimental glaucoma and retinal excitotoxicity. Invest Ophthalmol Vis Sci 49: 4515–4522. 18566471 10.1167/iovs.08-2078

[13] Goldenberg-Cohen N, Dadon-Bar-El S, Hasanreisoglu M, Avraham-Lubin BC, Dratviman-Storobinsky O, et al (2009) Possible neuroprotective effect of brimonidine in a mouse model of ischaemic optic neuropathy. Clin Experiment Ophthalmol 37: 718–729. 19788670 10.1111/j.1442-9071.2009.02108.x

[14] Lee KY, Nakayama M, Aihara M, Chen YN, Araie M (2010) Brimonidine is neuroprotective against glutamate-induced neurotoxicity, oxidative stress, and hypoxia in purified rat retinal ganglion cells. Mol Vis 16: 246–251. 20161817 PMC2822551

[15] Lambert WS, Ruiz L, Crish SD, Wheeler LA, Calkins DJ (2011) Brimonidine prevents axonal and somatic degeneration of retinal ganglion cell neurons. Mol Neurodegeneration 6: 4. 10.1186/1750-1326-6-4 PMC3035592 21232114

[16] Gao H, Qiao X, Cantor LB, WuDunn D (2002) Up-regulation of brain-derived neurotrophic factor expression by brimonidine in rat retinal ganglion cells. Arch Ophthalmol 120: 797–803. 12049586 10.1001/archopht.120.6.797

[17] Dong CJ, Guo Y, Wheeler L, Hare WA (2007) Alpha2 adrenergic receptor-mediated modulation of cytosolic Ca++ signals at the inner plexiform layer of the rat retina. Invest Ophthalmol Vis Sci 48: 1410–1415. 17325190 10.1167/iovs.06-0890

[18] Ozdemir G, Tolun FI, Gul M, Imrek S (2009) Retinal oxidative stress induced by intraocular hypertension in rats may be ameliorated by brimonidine treatment and N-acetyl cysteine supplementation. J Glaucoma 18: 662–665. 20010244 10.1097/IJG.0b013e31819c46b1

[19] Levkovitch-Verbin H, Harris-Cerruti C, Groner Y, Wheeler LA, Schwartz M, et al (2000) RGC death in mice after optic nerve crush injury: oxidative stress and neuroprotection. Invest Ophthalmol Vis Sci 41: 4169–4174. 11095611

[20] Oguni M, Tanaka O, Tamura H, Shinohara H, Kato K, et al (1995) Ontogeny of copper-zinc and manganese superoxide dismutase in the developing rat retina: immunohistochemical and immunochemical study. Ophthalmic Res 27: 227–233. 8539003 10.1159/000267710

[21] Fukui M, Zhu BT (2010) Mitochondrial superoxide dismutase SOD2, but not cytosolic SOD1, plays a critical role in protection against glutamate-induced oxidative stress and cell death in HT22 neuronal cells. Free Radic Biol Med 48: 821–830. 20060889 10.1016/j.freeradbiomed.2009.12.024 PMC2861908

[22] Larsson NG, Wang J, Wilhelmsson H, Oldfors A, Rustin P, et al (1998) Mitochondrial transcription factor A is necessary for mtDNA maintenance and embryogenesis in mice. Nat Genet 18: 231–236. 9500544 10.1038/ng0398-231

[23] Bonawitz ND, Clayton DA, Shadel GS (2006) Initiation and beyond: multiple functions of the human mitochondrial transcription machinery. Mol Cell 24: 813–825. 17189185 10.1016/j.molcel.2006.11.024

[24] Falkenberg M, Larsson NG, Gustafsson CM (2007) DNA replication and transcription in mammalian mitochondria. Annu Rev Biochem 76: 679–699. 17408359 10.1146/annurev.biochem.76.060305.152028

[25] Ngo HB, Kaiser JT, Chan DC (2011) The mitochondrial transcription and packaging factor Tfam imposes a U-turn on mitochondrial DNA. Nat Struct Mol Biol 18: 1290–1296. 22037171 10.1038/nsmb.2159 PMC3210390

[26] Xu S, Zhong M, Zhang L, Wang Y, Zhou Z, et al (2009) Overexpression of Tfam protects mitochondria against beta-amyloid-induced oxidative damage in SH-SY5Y cells. FEBS J 276: 3800–3809. 19496804 10.1111/j.1742-4658.2009.07094.x

[27] Hokari M, Kuroda S, Kinugawa S, Ide T, Tsutsui H, et al (2010) Overexpression of mitochondrial transcription factor A (TFAM) ameliorates delayed neuronal death due to transient forebrain ischemia in mice. Neuropathology 30: 401–407. 20102525 10.1111/j.1440-1789.2009.01086.x

[28] Piao Y, Kim HG, Oh MS, Pak YK (2011) Overexpression of TFAM, NRF-1 and myr-AKT protects the MPP(+)-induced mitochondrial dysfunctions in neuronal cells. Biochim Biophys Acta. 10.1016/j.bbagen.2011.08.007 21856379

[29] Yin W, Signore AP, Iwai M, Cao G, Gao Y, et al (2008) Rapidly increased neuronal mitochondrial biogenesis after hypoxic-ischemic brain injury. Stroke 39: 3057–3063. 18723421 10.1161/STROKEAHA.108.520114 PMC2726706

[30] Zhi Z, Cepurna W, Johnson E, Shen T, Morrison J, et al (2011) Volumetric and quantitative imaging of retinal blood flow in rats with optical microangiography. Biomed Opt Express 2: 579–591. 21412463 10.1364/BOE.2.000579 PMC3047363

[31] Tezel G, Chauhan BC, LeBlanc RP, Wax MB (2003) Immunohistochemical assessment of the glial mitogen-activated protein kinase activation in glaucoma. Invest Ophthalmol Vis Sci 44: 3025–3033. 12824248 10.1167/iovs.02-1136

[32] Schuettauf F, Rejdak R, Walski M, Frontczak-Baniewicz M, Voelker M, et al (2004) Retinal neurodegeneration in the DBA/2J mouse-a model for ocular hypertension. Acta Neuropathol 107: 352–358. 14745571 10.1007/s00401-003-0816-9

[33] Bosco A, Inman DM, Steele MR, Wu G, Soto I, et al (2008) Reduced retina microglial activation and improved optic nerve integrity with minocycline treatment in the DBA/2J mouse model of glaucoma. Invest Ophthalmol Vis Sci 49: 1437–1446. 18385061 10.1167/iovs.07-1337

[34] Wei MC, Zong WX, Cheng EH, Lindsten T, Panoutsakopoulou V, et al (2001) Proapoptotic BAX and BAK: a requisite gateway to mitochondrial dysfunction and death. Science 292: 727–730. 11326099 10.1126/science.1059108 PMC3049805

[35] Wasiak S, Zunino R, McBride HM (2007) Bax/Bak promote sumoylation of DRP1 and its stable association with mitochondria during apoptotic cell death. J Cell Biol 177: 439–450. 17470634 10.1083/jcb.200610042 PMC2064824

[36] Antonsson B, Conti F, Ciavatta A, Montessuit S, Lewis S, et al (1997) Inhibition of Bax channel-forming activity by Bcl-2. Science 277: 370–372. 9219694 10.1126/science.277.5324.370

[37] Schlesinger PH, Gross A, Yin XM, Yamamoto K, Saito M, et al (1997) Comparison of the ion channel characteristics of proapoptotic BAX and antiapoptotic BCL-2. Proc Natl Acad Sci U S A 94: 11357–11362. 9326614 10.1073/pnas.94.21.11357 PMC23466

[38] Desagher S, Martinou JC (2000) Mitochondria as the central control point of apoptosis. Trends Cell Biol 10: 369–377. 10932094 10.1016/s0962-8924(00)01803-1

[39] Oltvai ZN, Milliman CL, Korsmeyer SJ (1993) Bcl-2 heterodimerizes in vivo with a conserved homolog, Bax, that accelerates programmed cell death. Cell 74: 609–619. 8358790 10.1016/0092-8674(93)90509-o

[40] Yang E, Zha J, Jockel J, Boise LH, Thompson CB, et al (1995) Bad, a heterodimeric partner for Bcl-XL and Bcl-2, displaces Bax and promotes cell death. Cell 80: 285–291. 7834748 10.1016/0092-8674(95)90411-5

[41] Lai RK, Chun T, Hasson D, Lee S, Mehrbod F, et al (2002) Alpha-2 adrenoceptor agonist protects retinal function after acute retinal ischemic injury in the rat. Vis Neurosci 19: 175–185. 12385629 10.1017/s0952523802191152

[42] Vander Heiden MG, Chandel NS, Schumacker PT, Thompson CB (1999) Bcl-xL prevents cell death following growth factor withdrawal by facilitating mitochondrial ATP/ADP exchange. Mol Cell 3: 159–167. 10078199 10.1016/s1097-2765(00)80307-x

[43] Vander Heiden MG, Thompson CB (1999) Bcl-2 proteins: regulators of apoptosis or of mitochondrial homeostasis? Nat Cell Biol 1: E209–216. 10587660 10.1038/70237

[44] Beal MF (1995) Aging, energy, and oxidative stress in neurodegenerative diseases. Ann Neurol 38: 357–366. 7668820 10.1002/ana.410380304

[45] Mattson MP, Pedersen WA, Duan W, Culmsee C, Camandola S (1999) Cellular and molecular mechanisms underlying perturbed energy metabolism and neuronal degeneration in Alzheimer’s and Parkinson’s diseases. Ann N Y Acad Sci 893: 154–175. 10672236 10.1111/j.1749-6632.1999.tb07824.x

[46] Fan MM, Raymond LA (2007) N-methyl-D-aspartate (NMDA) receptor function and excitotoxicity in Huntington’s disease. Prog Neurobiol 81: 272–293. 17188796 10.1016/j.pneurobio.2006.11.003

[47] Ju WK, Kim KY, Angert M, Duong-Polk KX, Lindsey JD, et al (2009) Memantine blocks mitochondrial OPA1 and cytochrome c release and subsequent apoptotic cell death in glaucomatous retina. Invest Ophthalmol Vis Sci 50: 707–716. 18936150 10.1167/iovs.08-2499 PMC2678967

[48] Russo R, Cavaliere F, Berliocchi L, Nucci C, Gliozzi M, et al (2008) Modulation of pro-survival and death-associated pathways under retinal ischemia/reperfusion: effects of NMDA receptor blockade. J Neurochem 107: 1347–1357. 18803692 10.1111/j.1471-4159.2008.05694.x

[49] Chan SW, Nguyen PN, Ayele D, Chevalier S, Aprikian A, et al (2011) Mitochondrial DNA damage is sensitive to exogenous H(2)O(2) but independent of cellular ROS production in prostate cancer cells. Mutat Res 716: 40–50. 21843533 10.1016/j.mrfmmm.2011.07.019

[50] Ekstrand MI, Falkenberg M, Rantanen A, Park CB, Gaspari M, et al (2004) Mitochondrial transcription factor A regulates mtDNA copy number in mammals. Hum Mol Genet 13: 935–944. 15016765 10.1093/hmg/ddh109

[51] Ju WK, Misaka T, Kushnareva Y, Nakagomi S, Agarwal N, et al (2005) OPA1 expression in the normal rat retina and optic nerve. J Comp Neurol 488: 1–10. 15912498 10.1002/cne.20586 PMC1350956

